# Decision Analysis in the Management of Hip and Knee Osteoarthritis: A Systematic Review

**DOI:** 10.7759/cureus.83860

**Published:** 2025-05-10

**Authors:** Anser Daud, Jaskarndip Chahal, Jaryd Tong, David M Naimark, Daniel B Whelan, David Forner, Elad Apt, Graeme Hoit

**Affiliations:** 1 Division of Orthopaedic Surgery, University of Toronto, Toronto, CAN; 2 University of Toronto Orthopaedic Sports Medicine (UTOSM), Women's College Hospital, Toronto, CAN; 3 Temerty Faculty of Medicine, University of Toronto, Toronto, CAN; 4 Department of Nephrology, Sunnybrook Health Sciences Centre, Toronto, CAN; 5 University of Toronto Orthopaedic Sports Medicine (UTOSM), St. Michael's Hospital, Toronto, CAN; 6 Otolaryngology–Head and Neck Surgery, Dalhousie University, Halifax, CAN; 7 Division of Orthopaedic Surgery, Sunnybrook Health Sciences Centre, Toronto, CAN

**Keywords:** arthroplasty, health economic evaluation, knee osteoarthritis (koa), markov decision process, orthopaedic surgery, osteoarthritis of the hip, total hip arthroplasty (tha), total knee arthroplasty (tka)

## Abstract

Decision analysis is an increasingly used tool to guide policymakers and clinicians toward objective decision-making in uncertain clinical scenarios by analyzing the cost-effectiveness and health benefits of treatment modalities. In this systematic review, we summarize and appraise the current use of decision analysis in the management of hip and knee osteoarthritis (OA), the leading source of disability and societal costs in patients over 70. Publications involving decision analysis modelling for hip and knee OA between 1995 and 2021 were included. Among 54 included studies, there were 33 knee- and 18 hip OA-related models, while three were overlapping. Included articles primarily used Markov decision models (39), followed by simple decision trees (eight), microsimulations (five), and discrete event simulations (two) to compare OA treatment modalities. Models most commonly compared surgical procedures and devices (21), surgical versus nonoperative management (12), intraarticular injections (seven), and rehab therapies (five). More than half of all included studies (33) were published in the last five years. This study finds that there has been a large increase in the publication of hip and knee OA-related decision analysis models, particularly over the most recent five years. High-quality decision analysis models incorporate sensitivity and value of information analyses and take on broader, societal perspectives to incorporate utilities, direct costs, and indirect costs of management decisions. Surgeons should be familiar with the principles of decision analysis, which can be used to guide complicated real-world decision-making by incorporating risks and benefits of multiple strategies.

## Introduction and background

Hip and knee osteoarthritis (OA) is a leading cause of disability among older adults, with an estimated 300 million people affected worldwide [1-4]. Due to its widespread prevalence and chronic, progressive nature, there is a remarkable economic and societal burden associated with hip and knee OA [2,5,6]. This is of particular importance as adoption of new, expensive treatments may require diversion of funding from other programs within finite healthcare system budgets. Furthermore, discrepancies in health systems, societal and personal costs, and trade-offs between long-term outcomes and acute complications can complicate comparisons between treatment strategies. Although randomized controlled trials remain the gold standard for establishing evidence, their feasibility can be significantly limited by trial cost, follow-up, the number of trial arms, and difficulties in incorporating outcomes such as quality of life and out-of-pocket patient expenses.

Decision analysis is a systematic approach that often utilizes computer modeling to compare health outcomes and cost-effectiveness of various treatment modalities by incorporating evidence from a variety of sources [7-9]. When there are multiple strategies without a clearly superior choice, a decision analysis model can help determine the optimal intervention based on maximum benefit and minimum risks and costs [9].

In the context of OA, decision analysis models can enable clinicians, surgeons, payers, policymakers, and patients to make evidence-based decisions most appropriate for their respective lenses and outcomes of interest [7]. Decision analysis modeling is particularly applicable to OA, given that it is a widely prevalent condition involving a heterogeneous patient population and a diverse range of treatments. OA may be managed differently by various medical specialties and allied health disciplines, and there are varying timeframes that are relevant to a patient’s perspective. These factors can be incorporated into decision analysis models. Decision analysis models have frequently been used in drug-versus-drug economic evaluation studies, but are also well suited for the comparison of nonpharmacologic interventions.

As decision analysis continues to emerge as an increasingly utilized methodology, it is necessary that surgeons and other clinicians are familiar with its basic concepts and applications. This first-in-literature systematic review aims to summarize, appraise, and explain the current use of decision analysis in the nonpharmacologic management of hip and knee OA.

## Review

Methods

This systematic review is reported according to the Preferred Reporting Items for Systematic reviews and Meta-Analyses (PRISMA) guidelines [10] and was registered on the PROSPERO database (CRD42021229370).

Search Strategy and Study Selection

We conducted an English-language search of PubMed, Ovid MEDLINE, and Embase from 1995 until January 2021. The predefined search strategy was prepared using keywords related to decision analysis and hip and knee OA (Appendix A, Appendix B).

Nonduplicate titles and abstracts were reviewed by two reviewers independently (AD and GH) and included for full-text review if deemed relevant by either reviewer. Thereafter, full-text articles were assessed independently by two reviewers (AD and GH) for eligibility. Discrepancies were resolved by a combined review and consensus. While performing full-text extractions, citations were reviewed for additional eligible articles.

Study Eligibility Criteria

Inclusion criteria consisted of publications that used decision analysis to evaluate nonpharmacologic management strategies for hip and/or knee OA in humans. Exclusion criteria consisted of publications not using decision analysis-type modeling to suggest management options, animal studies, and publications involving only drug-versus-drug comparisons.

Data Collection and Synthesis

Two reviewers (AD and GH) independently extracted data in duplicate from the included studies, with discrepancies resolved by combined re-review and consensus. Variables including model type, perspective, inputs and data sources, costing data, discounting, time horizon, and outcomes were extracted. Data on model internal, external, and face validity, and methods of sensitivity analyses were also collected. Extracted data was analyzed qualitatively and with descriptive statistics where appropriate. These decision analysis concepts are defined in Appendix C, and examples of decision analysis model types refer to a previously published article by our group [11] for an explanation of model types.

Quality Review

Included studies were assessed for quality using the Consolidated Health Economic Evaluation Reporting Standards (CHEERS) statement [12]. This 24-point evaluation was developed to guide and assess decision analysis study quality.

Results

**Figure 1:**
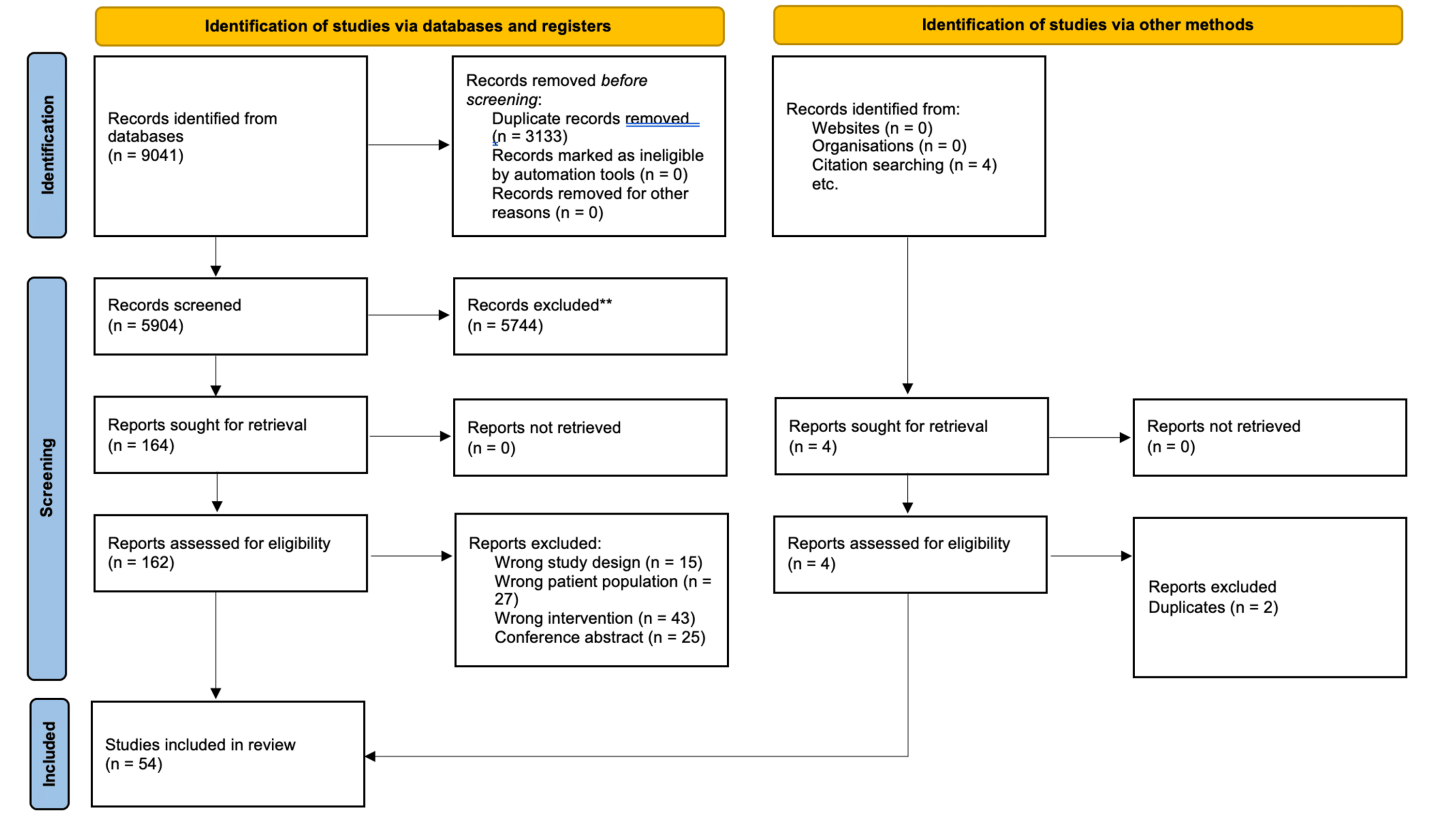
PRISMA flow diagram for new systematic reviews, which included searches of databases, registers, and other sources PRISMA, Preferred Reporting Items for Systematic reviews and Meta-Analyses

Our search yielded 5,908 unique records, of which 52 studies met the inclusion criteria. An additional two were identified from citations within included studies (Figure 1). A total of 33 studies focused on knee OA, 18 on hip OA, and three examined both hip and knee OA.

Study Characteristics

**Figure 2:**
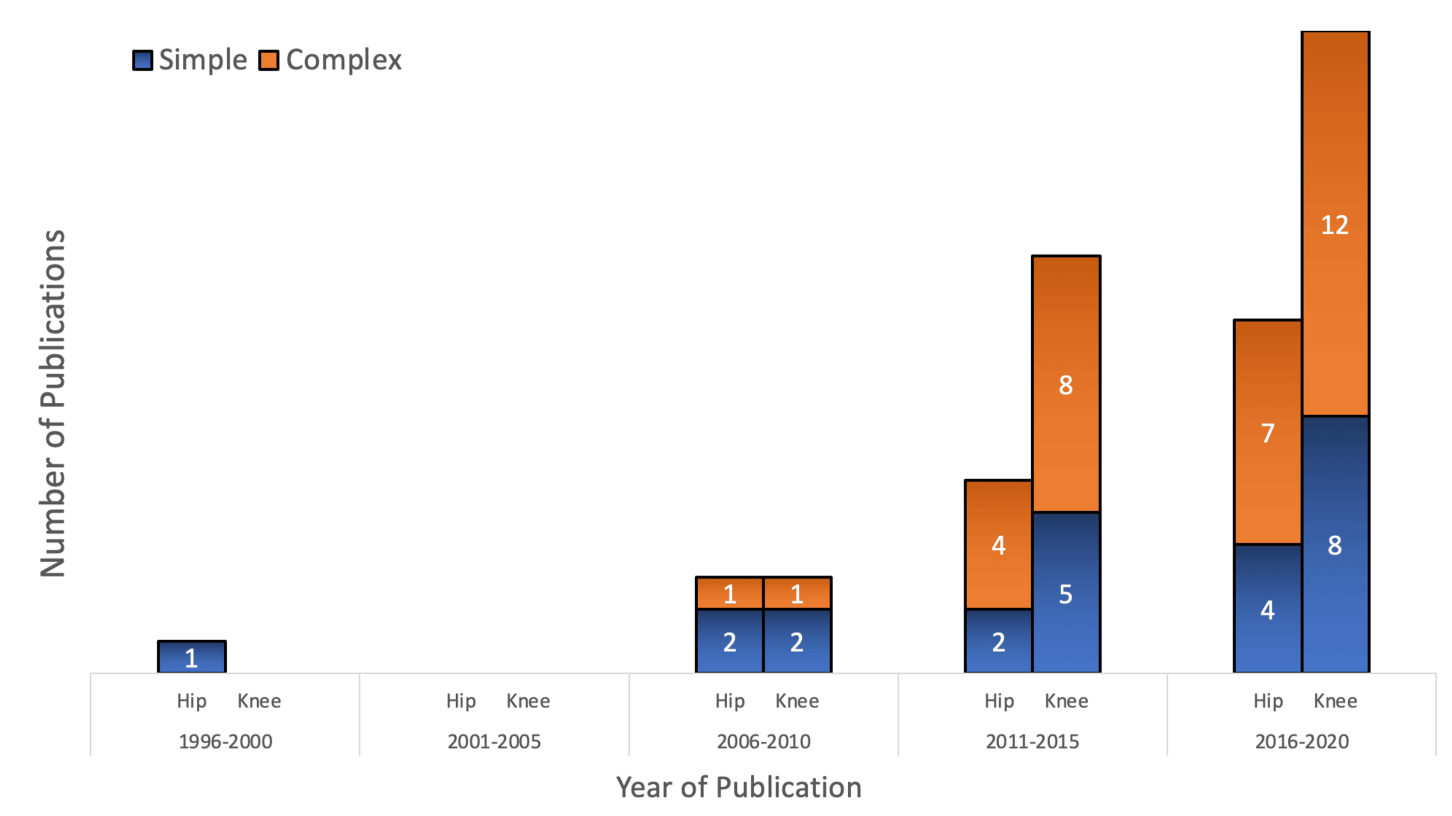
Frequency of simple and complex decision analysis publications in hip and knee OA over time Three studies encompassed both hip and knee OA (refer to Table 1). Models involving microsimulation, discrete event simulation, or probabilistic sensitivity analysis techniques were classified as “complex” in this figure (refer to Table 2). OA, osteoarthritis

The 54 included studies spanned from 1996 to 2020 and originated from 10 countries. The majority of studies were conducted in the United States (32 (59.2%)), followed by the United Kingdom (eight (14.8%)), Canada (five (7.4%)), The Netherlands (three (5.5%)), and Italy (two (3.7%)). Decision analysis utilization increased over time, with a total of 33 studies (61.1%) published between 2016 and 2020 (Figure 2). Studies were grouped according to indication (hip or knee OA) and by intervention or comparison (Table 1).

**Table 1:** Interventions and comparisons included in decision analysis models OA, osteoarthritis; THA, total hip arthroplasty; TKA, total knee arthroplasty

Joint	Intervention or comparison	Number of studies, n (%)
Knee (36)	TKA vs. alternate procedure	8 (14.8)
	TKA vs. nonoperative management	5 (9.3)
	Intraarticular injections	6 (11.1)
	Rehab therapies in knee OA	4 (7.4)
	Robotic or computer-assisted arthroplasty	4 (7.4)
	Evaluation of multiple therapies	2 (3.7)
	Meniscectomy vs. nonoperative	2 (3.7)
	Timing of TKA	2 (3.7)
	TKA surgical techniques	2 (3.7)
	Immediate vs. post-bariatric surgery TKA	1 (1.85)
Hip (20)	THA surgical techniques	7 (13.0)
	THA prostheses/devices	4 (7.4)
	THA vs. nonoperative	5 (9.3)
	Timing of THA	2 (3.7)
	Intraarticular injections	1 (1.85)
	Rehab therapies in hip OA	1 (1.85)

Model Characteristics

Five model types were used in the included studies (Table 2). Most commonly, studies used a discrete-time, discrete-state, or Markov cohort model (37 (68.5%)) [13-49], followed by a simple decision tree (eight (14.8%)) [50-57], discrete-time microsimulation (also known as first-order Monte Carlo) (one (1.9%)) [58], 2D simulation (2D-sim) (also known as a second-order Monte Carlo) (four (7.4%)) [59-62], and continuous-time discrete event simulation (two (3.7%)) [63,64]. Microsimulations, 2D-sims, and discrete event simulation models were found only in studies published from 2011 onward. Most models took on a health systems perspective (38 (68.5%)) [13-18,21,22,24,25,27,29,30,34,36-49,51-53,55-57,61,63-65], while fewer took on a wider societal perspective (16 (29.6%)), which incorporates additional costs not seen by the healthcare system (i.e., productivity losses and out-of-pocket expenses) [19,20,23,26,28,31-33,35,50,54,58-60,62,66]. Of the 16 models taking a societal perspective, all but one [50] were published after 2013.

**Table 2:** Characteristics of all included hip and knee OA decision analysis studies APM, arthroscopic partial meniscectomy; HA, hyaluronic acid; HTO, high tibial osteotomy; ICER, incremental cost-effectiveness ratio; OA, osteoarthritis; PAO, periacetabular osteotomy; PFA, patellofemoral arthroplasty; PT, physical therapy; QALY, quality-adjusted life year; RS, resurfacing; TDI, template-directed instrumentation; THA, total hip arthroplasty; THR, total hip replacement; TKA, total knee arthroplasty; TKR, total knee replacement; UKA, unicompartmental knee arthroplasty

Author and year	Location	Journal	Joint	Topic	Model	Model perspective	Target	Time horizon (year)	Outcomes	Currency	Costing data source	Quality score
Woods et al. (2017) [13]	York, UK	PLOS One	Knee	Adjunct nonpharmacologic interventions for knee OA	Markov	Health system	Policy/payer	0.154	Cost/QALY, ICER, productivity gain/loss	Euro	Literature review	17
van der Woude et al. (2016) [14]	Utrecht, The Netherlands	PLOS One	Knee	Knee joint distraction vs. TKA	Markov	Health system	Policy/payer	20	Cost/QALY, ICER	Euro	Dutch health insurance, hospitals, expert opinion	22
Cummins et al. (2009) [15]	Lebanon, USA	Journal of Bone and Joint Surgery	Hip	Antibiotic-impregnated cement vs. regular	Markov	Health system	Policy/payer	Lifetime	Cost/QALY, ICER	USD	National Inpatient Survey (US), literature review	21
Heintzbergen et al. (2013) [16]	Calgary, Canada	Value in Health	Hip	Metal on metal vs. conventional THA in young active patients	Markov	Health system	Policy/payer	15	Cost/QALY, ICER	CAD	Alberta Bone and Joint Health Institute patients	23
Busch et al. (2016) [17]	Nijmegen, The Netherlands	Hip International	Hip	Cemented vs. uncemented cup for THA in patients under 50	Markov	Health system	Policy/payer	15	Cost/QALY, ICER	Euro	Radboud University Medical Center, Dutch Guidelines to Costs in Medical Care	22
Carnes et al. (2016) [18]	Charlotte, USA	Journal of Bone and Joint Surgery	Hip	Ceramic vs. metal heads in THA	Markov	Health system	Policy/payer	Lifetime	Cost saving, net monetary benefit	USD	Premier Research database	20
Kunkel et al. (2018) [20]	Lebanon, USA	Arthroplasty	Hip	THA vs. nonoperative management in patients over 80	Markov	Societal	Policy/payer	30	Cost/QALY, ICER	USD	US CDC, literature review, Genworth Cost of Care Survey	23
Ponnusamy et al. (2018) [21]	London, Canada	Arthroplasty	Hip	THA vs. nonoperative management as a function of BMI	Markov	Health system	Policy/payer	15	Cost/QALY, ICER	USD	Literature review	24
Ponnusamy et al. (2018) [22]	London, Canada	Arthroplasty	Knee	TKA vs. nonoperative management as a function of BMI	Markov	Health system	Policy/payer	15	Cost/QALY, ICER	USD	Literature review	24
Premkumar et al. (2020) [23]	New York, USA	Arthroplasty	Hip	Bariatric surgery in combination with THA	Markov	Societal	Policy/payer	40	Cost/QALY, ICER, productivity gain/loss	USD	Literature review	21
Slover et al. (2008) [24]	Boston, USA	Journal of Bone and Joint Surgery	Knee	Value of computer navigation in high- vs. low-volume centers	Markov	Health system	Policy/payer	20	Cost/QALY, ICER	USD	Massachusetts General Hospital reimbursement data	17
Losina et al. (2009) [25]	Boston, USA	Archives of Internal Medicine	Knee	TKA vs. no TKA	Markov	Health system	Policy/payer	Lifetime	Cost/QALY, ICER	USD	Medicare reimbursement, literature review	24
Ruiz Jr et al. (2013) [26]	Rockville, USA	Journal of Bone and Joint Surgery	Knee	TKA vs. nonoperative management	Markov	Societal	Policy/payer and patient	Lifetime	Cost/QALY, ICER, productivity gain/loss	USD	Medicare inpatient claims	23
Mather et al. (2014) [27]	Durham, USA	BMC Musculoskeletal Disorders	Knee	TKA waiting periods	Markov	Health system	Policy/payer	Lifetime	Cost/QALY, ICER, net health benefit	USD	Medicare claims data, Medical Expenditures Survey	21
Bedair et al. (2014) [28]	Boston, USA	Journal of Bone and Joint Surgery	Knee	TKA in young vs. old	Markov	Societal	Policy/payer	30	Cost saving, productivity gain/loss	USD	Literature review	21
McLawhorn et al. (2016) [29]	New York, USA	Journal of Bone and Joint Surgery	Knee	Bariatric surgery prior to TKA	Markov	Health system	Policy/payer	Lifetime	Cost/QALY, ICER	USD	Centers for Medicare & Medicaid Services (medical services)	22
Chawla et al. (2017) [30]	New York, USA	The Bone & Joint Journal	Knee	PFA vs. TKA	Markov	Health system	Policy/payer	Lifetime	Cost/QALY, ICER	USD	UK National Joint Registry, Centers for Medicare & Medicaid Services, Healthcare Cost and Utilization Project, National Surgical Quality Improvement Program databases	21
Karmarkar et al. (2017) [31]	Baltimore, USA	Medical Care	Knee	Treatments for knee OA	Markov	Societal	Policy/payer	40	Cost saving, ICER, productivity gain/loss	USD	Literature review, Medical Expenditure Cost Survey	19
Kazarian et al. (2018) [32]	Philadelphia, USA	Journal of Bone and Joint Surgery	Knee	TKA, UKA, nonsurgical as a function of age	Markov	Societal	Policy/payer	Lifetime	Cost/QALY, ICER	USD	Literature review	22
Rongen et al. (2018) [33]	Nijmegen, The Netherlands	Osteoarthritis and Cartilage	Knee	Arthroscopic meniscectomy	Markov	Societal	Clinician	9	Cost/QALY, ICER, productivity gain/loss	Euro	Osteoarthritis Initiative database, literature review, Dutch healthcare authority	22
Yeroushalmi et al. (2022) [34]	New York, USA	Journal of Knee Surgery	Knee	Robotic-assisted UKA vs. traditional UKA	Markov	Health system	Policy/payer	5	Cost/QALY, ICER	USD	Medicare fee schedule	22
Rajan et al. (2020) [35]	Cleveland, USA	Journal of Bone and Joint Surgery	Knee	Platelet-rich plasma in combination with TKA	Markov	Societal	Clinician	Lifetime	Cost/QALY, ICER	USD	Medicare reimbursement schedule	22
Di Tanna et al. (2011) [36]	Rome, Italy	Journal of Surgical Research	Hip	Cementless vs. hybrid prostheses for THA	Markov	Health system	Policy/payer	Lifetime	Cost/QALY, ICER	Euro	Regional database in Italy	17
Pennington et al. (2013) [37]	London, UK	British Medical Journal	Hip	Cement vs. cementless vs. hybrid prosthesis for THA	Markov	Health system	Policy/payer	Lifetime	Cost/QALY, ICER, net monetary benefit	Euro	National Health Service Supply Chain, literature review	24
Pulikottil-Jacob et al. (2015) [38]	Coventry, UK	Bone and Joint Journal	Hip	Hip prostheses	Markov	Health system	Policy/payer	Lifetime	Cost/QALY	GBP	National Health Service Supply Chain, literature review	24
Pulikottil-Jacob et al. (2016) [39]	Coventry, UK	PLOS One	Hip	Metal-on-metal RS vs. traditional THA	Markov	Health system	Policy/payer and patient	10, lifetime	Cost/QALY, ICER	GBP	National Health Service Supply Chain, literature review	22
Migliore et al. (2019) [40]	Rome, Italy	ClinicoEconomics and Outcomes Research	Hip and knee	Viscosupplementation with Hylan G-F 20	Markov	Health system	Policy/payer	5	Cost/QALY, ICER	Euro	Italy’s National Health Service, literature review, pharmaceutical company	16
Smith et al. (2017) [41]	Norwich, UK	Knee Surg Sports Traumatol Arthroscopy	Knee	TKA vs. UKA vs. HTO	Markov	Health system	Policy/payer	10	Cost/QALY, ICER, net monetary benefit	GBP	Literature review	20
Burn et al. (2018) [42]	Oxford, UK	BMJ Open	Knee	UKA vs. TKA	Markov	Health system	Policy/payer	Lifetime	Cost/QALY, ICER	GBP	UK national tariff, Clinical Practice Research Datalink	24
Mari et al. (2016) [43]	Vandœuvre-lès-Nancy, France	Osteoarthritis and Cartilage	Knee	Early vs. late TKA	Markov	Health system	Policy/payer	Lifetime	Cost/QALY, ICER	Euro	French National Health Service, literature review	23
Mota (2013) [44]	Exeter, UK	Value Health	Hip	Early vs. late THA	Markov	Health system	Policy/payer	Lifetime	Cost/QALY, ICER	Euro	Literature review	20
Mujica-Mota et al. (2017) [45]	Exeter, UK	Orthopaedic Reviews	Hip	Timely vs. delayed THA	Markov	Health system	Policy/payer	Lifetime	Cost/QALY, ICER	Euro	Literature review	21
Clement et al. (2019) [46]	Edinburgh, UK	The Bone & Joint Journal	Knee	Robotic UKA vs. manual UKA/TKA	Markov	Health system	Policy/payer	Lifetime	Cost/QALY	GBP	Literature review, UK national tariff, manufacturer	20
Peersman et al. (2014) [47]	Antwerp, Belgium	The Knee	Knee	UKA vs. TKA	Markov decision analysis	Health system	Policy/payer	Lifetime	Cost/QALY, ICER	Euro	Belgian National Institute for Health and Disability Insurance	22
Moschetti et al. (2016) [48]	Boston, USA	Arthroplasty	Knee	Robotic UKA vs. manual UKA	Markov	Health system	Policy/payer	Lifetime	Cost/QALY, ICER	USD	Medicare Data, vendor financial recepits (MAKO Surgical Corp.)	21
Chang et al. (1996) [50]	Chicago, USA	Journal of American Medical Association	Hip	THA vs. nonoperative management	Decision tree	Societal	Policy/payer and clinician	Lifetime	Cost/QALY, ICER	USD	Literature review	22
Soohoo et al. (2006) [51]	Los Angeles, USA	Journal of Bone and Joint Surgery	Knee	UKA vs. TKA	Decision tree	Health system	Policy/payer and clinician	Lifetime	Cost/QALY, ICER	USD	Medicare 1998, MEDPAR	17
Meijer et al. (2015) [52]	New Orleans, USA	The Knee	Knee	Resurfacing patella	Decision tree	Health system	Policy/payer	5	Cost saving	USD	Medicare reimbursement schedule	16
Sharifi et al. (2008) [53]	San Francisco, USA	Journal of Bone and Joint Surgery	Hip	PAO vs. THA	Decision tree	Health system	Policy/payer, clinician, patient	30	Cost/QALY, ICER, net health benefit	USD	Institutional	23
Teng et al. (2019) [54]	Novena, Singapore	Journal of Public Health (Oxford)	Hip and knee	Hydrotherapy vs. land-based therapy	Decision tree	Societal	Policy/payer	0.25	Cost/QALY, ICER, productivity gain/loss	SGD	Hospital database	20
Hatoum et al. (2014) [55]	Chicago, USA	Journal of Medical Economics	Knee	Intra-articular bioengineered hyaluronic acid vs. conventional care	Decision tree	Health system	Policy/payer	1	Cost/QALY, ICER	USD	Medicare fee schedules, literature review	18
McLawhorn et al. (2015) [56]	New York, USA	Arthroplasty	Knee	TDI for TKA	Decision tree	Health system	Clinician	-	Cost saving	USD	Literature review	18
Rosen et al. (2020) [57]	New York, USA	Advances in Therapy	Knee	Intraarticular HA vs. conservative interventions	Decision tree	Health system	Policy/payer	0.5	Cost/QALY, ICER	USD	Literature review	10
Losina et al. (2015) [58]	Boston, USA	PLOS One	Knee	PT vs. PT + delayed APM vs. PT + immediate APM	Microsimulation	Societal	Policy/payer	10	Cost/QALY, ICER, `Loss	USD	Medicare fee schedules, red book online, healthcare cost and utilization, Bureau of Labour and Statistics, literature review	23
Nelson et al. (2014) [59]	Salt Lake City, USA	Arthritis Care Res (Hoboken)	Knee	Training rural providers to perform joint injections	Monte Carlo method	Societal	Policy/payer	0.5	Cost/QALY, ICER	USD	Bureau of Labor Statistics, Medicare national rate for Healthcare Common Procedure Coding System	22
Losina et al. (2019) [60]	Boston, USA	Arthritis Care Research (Hoboken)	Knee	Diet/exercise for obese/overweight knee OA patients	Monte Carlo method	Societal	Policy/payer	Lifetime	Cost/QALY, ICER, productivity gain/loss	USD	IDEA trial, Wake Forest personnel salaries	24
Smith et al. (2018) [61]	Boston, USA	Osteoarthritis and Cartilage	Knee	Health coaching and financial incentives to promote physical activity after TKR	Microsimulation	Health system	Policy/payer	Lifetime	Cost/QALY, ICER, net monetary benefit	USD	SPARKS trial, health logs	24
Silva et al. (2020) [62]	Boston, USA	Osteoarthritis and Cartilage	Knee	Physical activity in knee OA	Microsimulation	Societal	Policy/payer	3	Cost/QALY, ICER	USD	National Health and Nutrition Examination Survey, Medicare Beneficiary Survey, literature review	23
Castro et al. (2015) [63]	Bogota, DC, Colombia	Value in Health Regional	Knee	Viscosupplementation vs. conventional supportive therapy	Discrete event simulation	Health system	Policy/payer and clinician	5-20	Cost/QALY, ICER	USD	SISMED, Farmaprecios, SOAT tariff databases (Colombia)	19
Higashi and Barendregt (2011) [64]	Queensland, Australia	PLOS One	Hip and knee	THR and TKR in older Australians	Discrete event simulation	Health system	Policy/payer and patient	Lifetime	Cost/QALY, ICER	AUD	Health expenditure in Australia, Australian labor force statistics, literature review	24
Bozic et al. (2010) [65]	San Francisco, USA	Clinical Orthopaedics and Related Research	Hip	Metal-on-metal RS vs. traditional THA	Markov	Health system	Policy/payer and clinician	30	Cost/QALY, ICER, net monetary benefit	USD	Literature review	21
Konopka et al. (2015) [66]	Boston, USA	Journal of Bone and Joint Surgery	Knee	TKA vs. UKA vs. HTO	Markov	Societal	Policy/payer	Lifetime	Cost/QALY, ICER, net monetary benefit, productivity gain/loss	USD	Medicare reimbursement schedule	21
Koenig et al. (2016) [67]	Rockville, USA	Clinical Orthopaedics and Related Research	Hip	Societal benefits of THA	Markov	Societal	Policy/payer	Lifetime	Cost/QALY, ICER, net monetary benefit, productivity gain/loss	USD	Medicare reimbursement schedule, National Health Interview Survey	21

Studies were evaluated qualitatively to determine intended targets. With the exception of three models (5.5%) [33,35,56] which solely targeted clinicians, all others targeted policymakers and payers (51 (94.4%)). Of these 51, six (11.1%) also targeted clinicians [50,51,53,63,65] and four (7.4%) also targeted patients [26,38,53,64].

Models had varying time horizons, ranging from patient lifetime (27 (50%)), 30-40 years (six (11.1%)), 10-20 years (nine (16.7%)), 1-9 years (six (11.1%)), <1 year (four (7.4%)) or unspecified (one (1.9%)) (Table 2). Models defined willingness to pay (WTP) thresholds (the average price of one additional quality-adjusted life year (QALY)) at various amounts (converted to US dollars where appropriate) (Table 3).

**Table 3:** WTP thresholds WTP, willingness to pay

WTP threshold	Models, n (%)
≤$10,000	5 (9.4)
$20,000-$40,000	13 (24.5)
$50,000	25 (47.2)
$60,000-$80,000	6 (11.3)
100,000	12 (22.6)
≥$150,000	3 (5.7)
Unspecified	8 (15.1)

Model Inputs

Model inputs include utilities, probabilities, and costing data. Utility values were derived from patient-reported outcome data (indirect methods) (17 (31.5%)) [13,17,19,25,33,37-39,43,45,50,54,55,58,60,61,63], utility scores (direct methods) (24 (44.4%)) [16,18,20,23,24,26,27,29-31,34-38,41,46-48,51,56,57,59,65,66], both (11 (20.3%)) [14,15,21,22,32,42,45,49,53,62,64], or unspecified sources (two (3.7%)) [28,52]. In most cases, utility values were established from literature review (46 (85.2%)), and less often from arthroplasty databases (11 (18.5%)) [16-18,34-39,49,64], or author calculations (one (1.9%)) [26].

Within discrete-time decision models, the probability of certain events or health state transitions occurring must be input into the model. Most models obtained transition probability values from multiple sources, which consisted of literature (36 (66.7%)) [13,14,16,20,23,26-28,30,32,33,35,40,41,44,45,47,48,50-67], national/regional joint registries (20 (37.0%)) [14-18,24,28,30,34,36-39,42,44-47,49,67], national statistics or life tables (15 (27.7%)) [15,24-26,28,29,31,34,38,42-44,47,56,60], and institutional data (four (7.4%)) [17,18,21,22].

Costing data were obtained across a range of sources (Table 2), including literature review (26 (48.1%)), health administrative databases (16 (29.7%)), regional/national databases (13 (24.1%)), institutional data (nine (16.7%)), survey data (four (7.4%)), supply chain data (three (5.5%)), and expert opinion (one (1.9%)). Thirty-four studies denominated costs in US dollars, 11 in euros, five in United Kingdom pounds, and one study each in Australian, Canadian, and Singaporean dollars (Table 2). Costs and utilities were discounted at 1.5% in one study, 3-3.5% in 39 studies, 3.95-5% in seven studies, and were not applied in seven studies (Appendix C).

Model Outcomes

Forty-eight models (88.9%) reported QALYs as an outcome (Table 4). Disability-adjusted life years (DALYs) and revision-free life years (RFLYs) were reported in one model each. Cost per QALY/DALY/RFLY and incremental cost-effectiveness ratios were outcomes in 51 (94.4%) and 48 models (88.9%), respectively. Three models conducted a value of information (VOI) analysis [58,61,66]. Other model outcomes included loss of productivity (12 (22.2%)), net monetary benefit (8 (14.8%)), cost savings (4 (7.4%)), and net health benefit (2 (3.7%)).

**Table 4:** Outcomes measured in decision analysis models DALY, disability-adjusted life year; ICER, incremental cost-effectiveness ratio; QALY, quality-adjusted life year; RFLY, revision-free life year

Outcome	Number of studies, n (%)
QALYs	48 (88.9)
Cost/QALY (or DALY or RFLY)	51 (94.4)
ICER	48 (88.9)
DALYs	1 (1.85)
RFLYs	1 (1.85)
Cost saved	4 (7.4)
Net monetary benefit	8 (14.8)
Net health benefit	2 (3.7)
Productivity	12 (22.2)

Hip OA Topics

A range of topics were evaluated in the 20 included models focusing on hip OA (Table 1). Eighteen models evaluated total hip arthroplasty (THA) as at least one of the treatment options. Nine models compared THA versus another alternative, including nonoperative management (five) [20,22,50,64,67], hip resurfacing (three) [16,39,65], and periacetabular osteotomy in young patients (one) [53]. Five models evaluated THA implant types [18,36-38,49], and two models compared cementing techniques [15,17]. Of the remaining three models, two compared the timing of THA surgery [44,45] and one evaluated the cost-effectiveness of bariatric surgery in THA patients [23]. The final two hip OA models focused on comparing rehab therapies [54] and viscosupplementation against conventional supportive therapies [40].

Knee OA Topics

Of the 36 studies focusing on knee OA, 19 models were related to total knee arthroplasty (TKA) (Table 1). Of these, 14 studies compared TKA to at least one alternative treatment, including nonoperative care (five) [21,25,26,28,64], unicondylar knee arthroplasty (six) [32,41,47,51,66,68], high tibial osteotomy (one) [41], knee joint distraction (one) [14] and patellofemoral arthroplasty (one) [30]. Five models focused on technological advances within knee OA surgery by comparing conventional methods to robot-assisted surgery (three) [34,46,48], template-directed instrumentation (one) [24,56], and computer navigation (one) [24]. The final TKA study focused on comparing the cost-effectiveness of TKA with and without patellar resurfacing [52]. Two studies evaluated knee arthroscopy compared with nonoperative methods in OA patients [33,58]. The remaining studies focused on comparing nonoperative therapies, including intra-articular injections (six) [35,40,55,57,59,63] and rehab therapies (five) [13,54,60,62,69].

Internal and External Validity

Establishing internal and external validity is an important step in model development for decision analyses. Internal validity describes a systematic process of testing models to establish that intermediate outputs of the model, such as the failure rates or survival rates, reproduce those observed in real-world cohorts or populations of patients from which the model was developed (Appendix C). External validity involves comparison of model outputs to observed data that was not incorporated in model construction. All included studies exhibited face validity, with content experts in the authorship team. Only three studies (5.6%) reported efforts to confirm the internal validity of their models [33,55,64], while no included study reported measures to confirm model external validity.

An assessment of study discussions determined that 36 studies (66.7%) offered a literature review summarizing findings from other decision analysis publications. Among these studies, eight (14.8%) were able to contrast their findings with existing publications on the same topic [26,34,36,45,58,62-64].

Sensitivity Analysis

Sensitivity analyses are a mainstay of decision analysis and are used to demonstrate the robustness of the model result against changes to model inputs within reasonable estimate uncertainty (Appendix C). Overall, 50 models (92.6%) reported using sensitivity analyses, while four (7.4%) did not [18,31,46,50]. In total, 41 models (75.9%) used deterministic sensitivity analyses, and 24 models (44.4%) used probabilistic sensitivity analyses (Table 5).

**Table 5:** Utilization of sensitivity analysis methods in included decision analysis models

Sensitivity analysis	Number of studies, n (%)
One-way deterministic sensitivity analysis	8 (15.1)
Two-or-more-way deterministic analyses	4 (7.6)
Probabilistic sensitivity analysis	8 (15)
One-way deterministic and two-or-more-way deterministic analyses	8 (15.1)
One-way deterministic and probabilistic sensitivity analysis	8 (15.1)
One-way and two-or-more-way and probabilistic sensitivity analyses	13 (24.5)

Quality Assessment

The quality assessment of the included studies was evaluated as high, with the mean CHEERS score being 21 ± 2.7/24 points (range 10-24) (Table 2).

Discussion

To the best of our knowledge, this systematic review is the first to summarize and appraise the use of decision analysis in hip and knee OA. Results from this study demonstrate a trend of increased use of decision analysis models over the last two decades and that the complexity and quality of model type, testing, and reporting have evolved with time. Given this trend and the value decision analysis models provide, it is reasonable to expect continued use of these models to guide future management or policy questions. It is important for healthcare professionals to be familiar with decision analysis principles in order to keep up with current and future research.

We found that most decision analysis studies focused on hip and knee arthroplasty, though there was a range of important clinical and surgical topics. Other commonly investigated topics included the cost-effectiveness of various intra-articular injections, rehabilitation, surgical timing, and utilizing emerging technology (i.e., robotic surgery).

Within our review, decision analysis models included simple decision trees and Markov models as well as more advanced modelling techniques (i.e., microsimulations and discrete event simulations). Chang et al. published the first decision analysis related to hip OA in 1996 [50], which compared THA with nonoperative management utilizing a simple decision tree. This study can be used as a benchmark to demonstrate early decision analysis techniques and can be compared to later studies to showcase the evolution of model types, testing, and reporting. Microsimulation and discrete event simulation models, which allow for the incorporation of model memory and individual patient stochasticity and variation, appeared in the literature after 2011. Losina et al.’s study in 2015 [58] compared treatments for meniscal tears in the presence of knee OA and incorporated individual variability of patients within their model, built in uncertainty distributions around input parameters, and described the sensitivity analyses used to arrive at their results, which included VOI analyses (Appendix C). This sophisticated microsimulation model, which clearly specifies model components, is an example of the evolution of decision analysis since Chang et al.’s study in 1996 [50]. In keeping with modelling guidelines [70], the Journal of the American Medical Association User’s Guide to evaluating decision analysis [71,72], and the CHEERS quality evaluation (24/24 points).

Despite their variations in complexity, all decision analysis models follow similar principles [7,9]. They require specifying a clinical scenario within which a decision must be made regarding alternative strategies where potential outcomes are uncertain, identifying the relevant patient population, selecting the time frame of analysis, estimating probabilities of experiencing health events, or specifying time-to-event distributions, and associated pay-offs [7]. Few studies performed VOI analyses (three), which allow for the calculation of the value of reducing the uncertainty around model input parameters. This is often employed to determine if conducting a study to provide additional precision around an outcome (i.e., complication rates of a new implant) is cost-justifiable, based on the ability of that information to change the result of the model. As research grant funding becomes increasingly scarce [73], the use of VOI analyses in this way may become more popular as a means to prioritize studies that provide the most potential benefit per dollar invested.

Two important markers of decision analysis study quality include sensitivity analyses and validity testing [70]. Of the included studies, 49 incorporated sensitivity analysis to test the robustness of their conclusions, with recent models more often utilizing probabilistic and multi-way analyses (Figure 2). Of the studies included, three studies [33,55,64] reported testing internal validity, yet none described the specifics of their methods. No studies within this review reported external validity testing, representing the potential for continued evolution of decision analysis modeling within hip and knee OA, especially with the increasing prevalence of large arthroplasty registries. Investigators of future decision analysis studies should focus on demonstrating the validity of their models.

The outcomes of decision analyses are frequently used in the context of healthcare resource allocation as well as determining the value of different clinical strategies [74,75]. Thus, framing and reporting of study outcomes to target different stakeholders, such as clinicians and policymakers, is often decided by the study investigators. We found that a majority of included models targeted payers and policymakers (42), suggesting that decision analysis models, which are clinician- and patient-centered, are not yet commonly employed to resolve hip and knee OA treatment strategies. Furthermore, most models took on a health system perspective (37) for establishing net costs rather than a societal perspective, which incorporates direct and indirect costs as well as outcomes in a broader sense (i.e., loss of personal productivity). However, nearly all models utilizing a societal perspective were published after 2013. This may represent a trend toward acknowledging and incorporating a broader lens in evaluating hip and knee OA interventions. We anticipate the future of decision analysis in hip and knee OA to increasingly utilize advanced modelling techniques such as microsimulation and discrete event simulation while incorporating a wider, societal perspective more frequently.

Limitations

This review has several limitations. First, despite best efforts to search multiple online libraries, all studies utilizing decision analysis in hip and knee OA are not presumed to be included. Publication bias toward decision analysis models with good performance and/or those of higher quality means that there are likely some previously conducted studies that were not found by our systematic search because they did not reach publication. Given the size of our review and the detail of our data extraction, we opted not to perform a gray literature search of conference abstracts. This may have falsely biased the results of our quality assessment toward higher quality and reporting scores. Second, the quality assessment of the included articles using the CHEERS guidelines is somewhat subjective, and there are no records of inter- or intra-rater reliability of this score, which could lead to some variation should this exercise be repeated by another group. Lastly, we made the decision to focus on decision analyses that incorporated interventions beyond simple drug-drug comparisons, given that the target audience of our study was surgeons. This led to the exclusion of some well-conducted decision analyses focusing on hip and knee OA from a pharmacological treatment perspective.

## Conclusions

Decision analysis models are powerful tools that facilitate the comparison of the quality of life and the cost associated with diagnostic and management options, leading to evidence-based decisions. Models are increasingly found in hip and knee OA literature, with increasing levels of complexity to incorporate broader, societal perspectives, particularly over the last five years. This review highlights variability in modelling practices, outcomes, and quality. In particular, the highest quality models incorporate sensitivity and VOI analyses, validity testing, and take on broader societal perspectives to incorporate direct costs to the system as well as indirect costs to the patient. Given the immense potential to aid clinical decision-making, surgeons and other clinicians should be familiar with the principles of decision analysis so that they may offer evidence-based recommendations to patients that incorporate utilities as well as costs of a decision.

## Appendices

Appendix A

**Table 6:** Search terms used in Ovid MEDLINE search strategy

#	Search term or keyword
1	exp Osteoarthritis/	
2	exp Decision Support Techniques/
3	exp "Costs and Cost Analysis"/
4	exp Decision Theory/ or exp Decision Making/
5	exp Decision Trees/
6	exp Models, Statistical/ or exp Markov Chains/ or markov.mp. or exp Cost-Benefit Analysis/
7	exp Models, Economic/
8	decision analysis.mp.
9	decision model*.mp.
10	discrete event simulation.mp.
11	microsim*.mp.
12	2 or 3 or 4 or 5 or 6 or 7 or 8 or 9 or 10 or 11
13	1 and 12
14	exp animals/ not (exp animals/ and exp humans/)
15	13 not 14
16	limit 15 to english language
17	limit 16 to humans
18	limit 17 to yr="1995 -Current"

Appendix B

**Table 7:** Search query used in PubMed search performed in February 2021

#	Search term or keyword
1	(((((osteoarthritis[MeSH])
2	AND ((decision support techniques[MeSH])
	OR ("Costs AND Cost Analysis"[MeSH])
	OR ("Decision Theory"[mesh])
	OR ("Decision Making"[mesh])
	OR ("Decision Trees"[mesh])
	OR ("Markov Chains"[MeSH])
	OR ("Models, Economic"[mesh])
	OR ("Models, Statistical"[mesh])
	OR ("Cost-Benefit Analysis"[mesh])
	OR (decision analysis[Title/Abstract])
	OR (decision model*[Title/Abstract])
	OR (discrete event simulation[Title/Abstract])
	OR (microsim*[Title/Abstract])))
3	NOT (("animals"[mesh])
4	AND ("english"[lang])) AND "humans"[mesh])

Appendix C: Definition of decision analysis terms and concepts

The aim of this appendix is to define terms that relate to decision analysis that are included in the main article.

Decision analysis model types: For conceptual specifics and examples of decision analysis model types, we suggest referring to a previously published article by our group (doi: 10.1177/0194599820957288).

Model perspectives: Models must take on a particular perspective in order to determine which costs and benefits will be included in the model and analysis. Common perspectives include those taken from a health system payer perspective and a societal perspective. Models that are made from a health system payer perspective will typically include only costs directly to the health system of interest (i.e., surgical costs or hospital stays). Societal perspective models may incorporate additional costs which are felt by society or the individual patient, such as missed time off work, parking and commuting costs, and health expenses not covered by the payer system.

Willingness to pay (WTP) threshold: Prior to conducting the analysis, it is best practice for the investigators to set an a priori cost-per-benefit amount they will consider to be cost-effective in the event that one comparator provides a health benefit at additional cost. This is the WTP threshold. Certain jurisdictions have declared WTP thresholds (i.e., $50,000/quality-adjusted life year (QALY)) that are considered cost-effective and are commonly used for the purposes of funding new health technologies. Investigators may examine the intervention across a variety of WTP thresholds to determine at which point a transition is made from a technology being considered cost-effective to cost-ineffective.

Time horizon: Most decision analysis models will continue to accumulate costs and benefits until a certain simulated time point is reached - this is the time horizon. Investigators must set the time horizon a priori, at which point the accumulated differences between interventions will be compared, similar to comparing outcomes at a predetermined clinical follow-up time point in clinical research. The time horizon can be a discrete point (i.e., 10 years) or based on accumulated events (i.e., all subjects have died).

Discounting: Health benefits and costs realized in the future are worth less than those realized immediately. The amount to which these values depreciate is influenced by the audience. Societal values and inflation rates are factored into determining appropriate discounting. Incremental utilities and costs are typically discounted at 1.5-3% per year, and there are often conventional discount rates for different countries and regions.

Model Input Parameters

All decision analysis models require inputs, which can be sourced from primary research, previously reported literature, particular datasets or databases, or “assumed” based on expertise and best available estimates. Investigators should disclose how they arrived at each input, and when sourcing from the literature, if a systematic method was applied to find sources of evidence and arrive at the estimates used. In the event that an inputted value is inexact or may vary, attempts can be made by investigators to incorporate variance through drawing estimates from distributions, allowing stochasticity (see Uncertainty) and conducting sensitivity analyses across ranges.

Utilities: A health utility is a number that describes a particular health state by assigning it a value so that it can be compared to other discrepant states of health. Two popular forms of utility measurement are the QALY and the disability-adjusted life year. Values range from 0 (death) to 1 (perfect health) and can accumulate over time. Various methods are used to determine the appropriate utility value for each condition (see direct and indirect methods), and these can vary with different societies and cultures. Utility values must be assigned within each model for the health states examined in order to compare the health outcomes across different health technologies.

Direct methods: Direct methods of determining health utility values require subjects to map their preferences for different health states onto a utility scale either by use of a visual analogue scale or by a trade-off experiment (i.e., standard gamble or time trade-off). Investigators may use direct methods themselves with real-life patients to determine the utility values they input or use the values from relevant literature that conducted patient-level studies.

Indirect methods: Indirect methods involve quantifying health utility by comparing the scores of those in different health states to healthy volunteers from a quality of life patient-reported outcome measure (most commonly the EQ-5D). These scores are then converted through validated equations into health utility. Investigators may use indirect methods themselves with real-life patients to determine the utility values they input or use the values from relevant literature that conducted patient-level studies.

Probabilities: The chances of different events occurring to subjects within the model must be inputted by investigators. These probability values are often sourced from literature or primary research and standardized to the cycle length of the model. Depending on the model type, probabilities can be as simple as a percent chance or as complex as multivariable equations specific to individual patients traversing through the model.

Costs: Costs are often accumulated over time within decision analysis models in order to make cost or cost-effectiveness comparisons between different health technologies. Costs inputted into the model are commonly sourced directly (i.e., from the hospital financial departments or device companies) or from literature. Costs should be standardized to one currency in one year (i.e., 2020 USD) and inflation should be accounted for by use of the appropriate inflation indices.

Uncertainty

First-order uncertainty: The costs and benefits may differ from patient to patient in a model due to both stochastic effects and/or differences in individual patient-level characteristics. This can be explored using microsimulation models.

Second-order uncertainty: Costs and benefits may differ due to variation in underlying parameters. This can be explored within models using probabilistic sensitivity analyses.

Two-dimensional simulations can be utilized to explore both first and second-order uncertainty.

Model Outcomes

Incremental cost effectiveness ratios (ICERs): ICERs combine the incremental difference in both cost and effectiveness (QALYs) between two different technologies into one single ratio that can be compared ([cost1-cost2]/[QALE1-QALE2]). The incremental cost-effectiveness can also be plotted on a quadrant plane. Interventions that provide increased effectiveness but have increased cost, or those that decrease cost but have decreased effectiveness are evaluated in the context of a WTP threshold to determine if they are indeed cost-effective in comparison to the alternative. Both of these scenarios would produce ICERs >0. Technologies that have decreased benefit and increased cost are “dominated” by their comparator, and those that have increased benefit and decreased cost are dominant. Both of these scenarios produce ICERs <0.

Net monetary benefit (NMB): To calculate NMB, effectiveness is assigned a monetary value (i.e., $30,000 per additional QALY) and combined with cost in order to arrive at a single number for comparison of cost effectiveness. NMBs for different technologies can be easily compared since they are expressed simply as a currency value. This is termed the incremental NMB.

Net health benefit (NHB): Similarly to NMB, NHB also expresses both cost and benefit in terms of a singular unit (typically QALYs) by converting costs into benefit values by a set ratio (i.e., -1 QALY per additional $30,000 in cost). NHBs can be compared directly between technologies, which is termed the incremental net benefit health.

Cost-effectiveness acceptability curves (CEAC): This is a graphical representation that plots the percentage of cost-effective iterations of the comparator technology in the model on the y-axis against varied WTP thresholds on the x-axis. Any percentage greater than 50% would be considered cost-effective at that given WTP threshold.

Value of information analysis (VOI): VOI analyses are conducted to quantify the value expected from reducing the uncertainty around a particular parameter of interest through further study and data collection. Practically, these analyses are often conducted to determine whether the cost of conducting a research trial is reasonable given the expected information to be gained.

Validation

Model validation consists of comparing model outputs to real, observed data. There are two forms of model validation, internal and external.

Internal validity: This uses real data that was used to create the model to ensure that the model is producing results that reflect truth. Typically, the main outcomes of decision analysis models are costs, effectiveness estimates, and cost-effectiveness. However, other by-product outputs (i.e., the percentage of patients that progressed from one disease state to another, the number of deaths, etc.) can be compared to the same outcomes from the studies used for the source data to ensure the model is a valid representation of the health states and technologies examined.

External validity: This uses real data that was not used to create the model to compare outcomes and determine whether the model is valid to other datasets. Commonly, investigators may choose to not utilize one particular landmark study or a well-known data bank when arriving at their input parameters. The excluded study/databank will then be used to compare to model outputs for the purpose of external validation.

Sensitivity Analyses

Within the context of decision analyses, sensitivity analyses are conducted to determine how the changing parameter inputs across plausible values can affect the outcomes of the model.

One way: A one-way sensitivity analysis varies an individual parameter across a plausible range with all other variables maintaining their base-case specifications.

Two-way and multi-way: A multi-way sensitivity analysis varies two or more parameters across their plausible ranges at the same time. Representing more than two varied parameters at a time becomes very complex and is typically performed by breaking the analyses into difference scenarios (i.e., by varying implant cost and complication rate at the same time for different age groups of patients separated by decade).

Deterministic: Deterministic sensitivity analyses provide concrete but potentially biased answers as to the cost-effectiveness of different health technologies across ranges of variables. From this type of analysis, one may conclude that Novel Technology X is cost-effective as long as the complication rate remains lower than Y.

Probabilistic: Probabilistic sensitivity analyses are a way of building in and describing the uncertainty around these conclusions by incorporating distributions of the variables being modelled and varied. From this type of analysis, one may conclude that Novel Technology X has a 60% chance of being cost-effective at complication rate Y. Due to its popularity and increased complexity from deterministic analyses, the probabilistic sensitivity analysis is sometimes regarded as a model type in itself.
